# The complete mitochondrial genome sequence of *Boechera stricta*

**DOI:** 10.1080/23802359.2018.1501323

**Published:** 2018-08-09

**Authors:** Junji Li, Changwei Bi, Jing Tu, Zuhong Lu

**Affiliations:** a State Key Laboratory of Bioelectronics, School of Biological Science and Medical Engineering, Southeast University, Nanjing,China;; b MIIT Key Laboratory of Advanced Display Materials and Devices, School of Environmental and Biological Engineering, Nanjing University of Science and Technology, Nanjing, China

**Keywords:** Boechera stricta, mitochondrial genome, phylogeny

## Abstract

*Boechera stricta* (*B. stricta*) is a wild relative of Arabidopsis, occurring in mostly montane regions of western North America. In this article, we assembled the complete mitochondrial (mt) DNA sequence of *B. stricta* into a circular genome of length 271,601 bp, including 31 protein-coding genes, 21 tRNA genes, and 3 rRNA genes. From the neighbour-joining phylogenetic tree was constructed, based on the 23 conserved protein-coding genes of *B. stricta* and other 23 plant species, and the phylogenic relationship and evolution position of *B. stricta* were determined. The complete mt genome would be useful for further investigation of the genotype-by-environment interactions in mitochondria of *Boechera*.

*Boechera stricta*, a close relative of the model plant *Arabidopsis thaliana*, mostly grows in montane areas of western North America. It is an important plant to study the species’ evolution in environments (Wagner et al. 2014) according to the genetic diversities introduced by self-fertilization of wild accessions (Schranz et al. 2007). In a recent study, a major chromosomal inversion in the *Boechera stricta* genome was identified to control ecologically important traits during the incipient speciation (Lee et al. 2017). However, the complete mitochondrial genome of *Boechera stricta*, which encodes the main generation of cellular ATP and plays a predominant role in metabolic regulation, has not been reported until now. Hence in this study, we assembled and primarily analyzed the entire mitochondrial genome sequence of *Boechera stricta*.

The *Boechera stricta* sample used in this study was harvested from a contact zone of its two differentiated subspecies in the northern Rocky Mountains (Geographic coordinate: 45°41′26″ N, 113°54′29″ W). The genomic DNA was extracted from etiolated seedlings according to the reference (Lee, Wang et al. 2017), and then was deposited in Department of Energy Joint Genome Institute (CA, USA). In this article, we assembled the complete mitochondrial DNA sequence of *Boechera stricta* into a free-of-gap genome of 271,601-bp length using the software of Newbler v3.0 from 454 GSFLX sequencing data (Wang et al. 2018), and then revised it with more abundant sequencing data from Illumina 2500 sequencing platform (Bi et al. 2016). Finally, the complete mt-genome sequence was submitted to GenBank with the accession number of MH545496. We calculated that the GC content of this mitochondrial genome to be 44.93% (A: 27.53%, C: 22.38%, G: 22.55% and T: 27.54%), which is a common value in Brassicaceae.

We used MITOFY program to annotate genes in the mitochondrial genome of *Boechera stricta* (Alverson et al. 2010), and identified 55 genes, including 31 protein-coding genes, 21 tRNA genes, and 3 rRNA genes. Among these, the trnK-TTT gene contains two copies, trnY-GTA contains three copies, and trnS contains four copies. All the protein genes have an ATG start codon except for the *nad1* gene with ACG as its initiation codon and the *mttB* gene with an undetermined start codon. Additionally, we found four types of stop codons in the protein-coding genes: TAA (17 genes: *atp4, atp6, cox1, cox2, ccmB, ccmc, ccmFc, nad1, nad2, nad3, nad4L, nad5, nad6, nad9, rpl5, rpl6,* and *rps4*), TGA (8 genes: *atp8, atp9, ccmFn, cob, cox3, nad4, rpl2,* and *rps12*), TAG (5 genes: *atp1, matR, mttB, nad7* and *rps3*) and one undetermined stop codon in *sdh4*. Furthermore, a total number of 32 exons and 18 introns were identified in nine protein-coding genes (*rps3, cox2, nad1, nad2, nad4, nad5, nad7, ccmFc* and *rpl2*). In order to clarify the position of *Boechera stricta* in the phylogenetic tree, twenty three conserved protein-coding genes, including *atp1*, *atp4*, *atp6*, *atp8*, *atp9*, *ccmB*, *ccmC*, *ccmFc*, *ccmFn*, *cob*, *cox1*, *cox2*, *cox3*, *matR*, *nad1*, *nad2*, *nad3*, *nad4*, *nad4L*, *nad5*, *nad6*, *nad7,* and *nad9*, were selected from 24 plant mitochondrial genomes. As shown in Figure 1, the neighbor-joining tree demonstrated that *Boechera stricta* was evolutionarily close to *Brassica napus* than *Arabidopsis thaliana,* all belongs to Brassicaceae.

**Figure 1.**
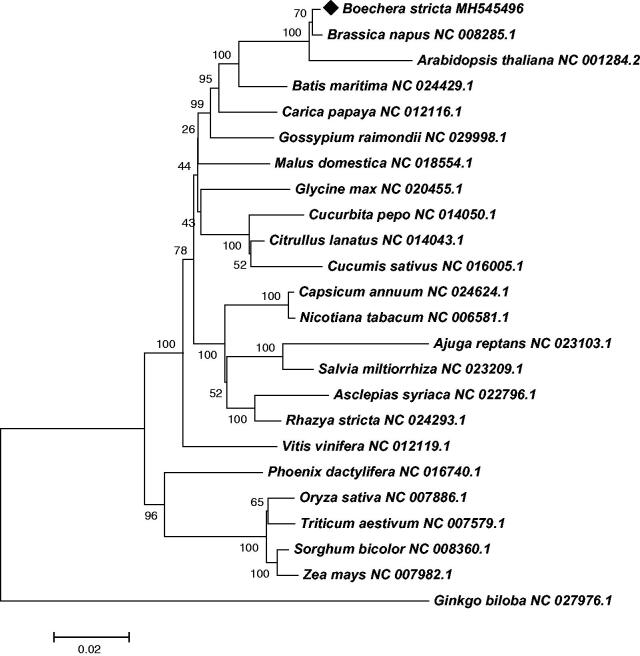
The Neighbor-Joining phylogenetic tree of 24 plant mt genomes based on 23 conserved mitochondrial genes. Bootstrap values are listed for each node. Accession numbers for tree construction are listed right to their scientific names.
